# Functional conservation of IR75q.2 in the recognition of volatile acids and aldehydes in two *Spodoptera* species

**DOI:** 10.1007/s44297-026-00082-7

**Published:** 2026-07-21

**Authors:** Jin-Meng Guo, Ji-Xiang Wang, Yu He, Xuan-Pu Luan, Zhi-Qiang Wei, Xiao-Long Liu, George F. Obiero, Qi Yan, Shuang-Lin Dong, Jin Zhang

**Affiliations:** 1 Sanya Institute of Nanjing Agricultural University, Nanjing Agricultural University, Nanjing, China; 2 State Key Laboratory of Agricultural and Forestry Biosecurity, College of Plant Protection, Nanjing Agricultural University, Nanjing, China; 3 Key Laboratory of Integrated Management of Crop Disease and Pests (Ministry of Education), Nanjing Agricultural University, Nanjing, China; 4 Key Laboratory of Soybean Disease and Pest Control (Ministry of Agriculture and Rural Affairs), Nanjing Agricultural University, Nanjing, China; 5 Department of Biochemistry and Biotechnology, The Technical University of Kenya (TU-K), Nairobi, Kenya

**Keywords:** Ionotropic receptor, Phylogenetic analysis, IR75 clade, *Xenopus* oocyte expression, *Spodoptera*, Molecular docking

## Abstract

Ionotropic receptors (IRs) serve as important chemosensory receptors for the perception of diverse ecological volatiles in insects, but their specific functions in Lepidoptera remain largely unexplored. This study systematically compared the ligand recognition profiles and key binding residues of the highly expressed IR75q.2 receptor in two *Spodoptera* species. Phylogenetic and conserved motif analyses revealed sequence and structural conservation of the IR75 receptors across five noctuid species. Transcriptome data analysis further showed that IR75q.2 had the highest expression in the antennae among the clade, suggesting its importance in olfaction. Functional assays using the *Xenopus* oocyte expression system showed that IR75q.2 from two *Spodoptera* species displayed conserved ligand response profile, responding to three medium-chain fatty acids and their corresponding aldehydes, with nonanoic acid as the strongest ligand. Molecular docking and site-directed mutagenesis further identified Phe284/285 and Arg289/290 as conserved key residues involved in nonanoic acid recognition by IR75q.2. Finally, nonanoic acid was determined to elicit both electrophysiological and behavioral responses in adults of the two *Spodoptera* species. Taken together, our study reveals conserved ligand recognition profiles and key binding residues of IR75q.2 in two *Spodoptera* species, providing insights into the functional evolution of IR75 clade in the olfactory system.

**Supplementary Information:**

The online version contains supplementary material available at 10.1007/s44297-026-00082-7.

## Introduction

Insects rely on chemosensory systems to perceive environmental chemical cues that regulate key behaviors, including host selection, predator avoidance, mating and oviposition [1–4]. These processes involve multiple chemosensory proteins, including odorant binding proteins (OBPs) [5], gustatory receptors (GRs) [6], odorant receptors (ORs) [7], ionotropic receptors (IRs) [8], sensory neuron membrane proteins (SNMPs) [9] and odorant degrading enzymes (ODEs) [10]. Among these, ORs have been extensively characterized as ligand-gated ion channels responsive to a broad range of odorants [11]. In addition to ORs, insects possess IRs, a distinct receptor family derived from ionotropic glutamate receptors (iGluRs), which are involved in detecting volatile acids, amines, and non-olfactory cues such as taste, humidity and temperature [8, 12–17].

Recent progress in molecular biology has improved our understanding of IR functions in insects, particularly in dipteran species such as fruit flies and mosquitoes [8, 18–21]. Studies in these species have shown that coreceptors such as IR8a and IR25a are crucial for detecting various volatile organic compounds in their environment [18, 22, 23]. Similar to their ancestral iGluRs, these coreceptors exhibit complex structures, including an extensive extracellular amino-terminal domain (ATD) and a ligand binding domain (LBD). The LBDs comprise two subdomains (S1 and S2) that form a ‘Venus flytrap’ structure around an ion channel pore. Functionally, IRs assemble into tetrameric complexes, consisting of a mandatory coreceptor (IRco, either IR8a or IR25a) and up to three odor-specific partners (designated IRx, e.g. IR75q.2 in this study) [24, 25]. Although an increasing number of IR-coding genes has been identified in lepidopteran genomes [26–28], detailed functional studies remain limited. This knowledge gap limits our understanding of the roles of IRs in the evolution of insect olfactory systems and hampers the development of molecular-targeted pest control strategies.

IRs are broadly categorized into antennal IRs (A-IRs) and divergent IRs (D-IRs). A-IRs are mainly expressed in antennal olfactory sensory neurons (OSNs) and are involved in detecting a broad range of odorants [17, 29, 30]. In contrast, D-IRs are distributed across multiple sensory organs and are associated with non-olfactory functions [12, 15, 20, 31–37]. Within the A-IRs, the IR75 clade has been identified in multiple insect lineages, particularly Diptera [38, 39] and Lepidoptera [26]. IR75s are highly diverse and have undergone lineage-specific expansion, suggesting adaptation to ecological niches and sensory modalities. Recent studies have linked IR75s to the detection of fatty acids and aldehydes, key chemical cues guiding insect behaviors [38–42]. However, the molecular mechanisms and evolutionary dynamics of IR75s in non-dipteran species, particularly Lepidoptera, remain poorly understood. Although IR75q.2 has been functionally characterized in *Spodoptera frugiperda* [40], whether its ligand recognition profile is conserved across other *Spodoptera* species remains unclear. In addition, the structural basis of ligand recognition and the key residues involved have not yet been characterized in these species.

In this study, we systematically compared IR75q.2, a member of the IR75 clade, in two *Spodoptera* species (*S. litura* and *S. exigua*). Both species are major agricultural pests with a broad host range and high dispersal ability [43, 44]. By integrating phylogenetic analysis, expression profiling, *Xenopus* oocyte functional assays, molecular docking, and site-directed mutagenesis, we examined whether IR75q.2 exhibits conserved ligand recognition profiles and key binding residues in the two species. Our results revealed conservation of IR75q.2 between these two *Spodoptera* species in gene structure, amino acid sequence, tissue expression, ligand response profile and key residues involved in ligand recognition, providing a comparative framework for understanding the functional genetics and evolution of the IR75 subfamily in Lepidoptera.

## Results

### IR75 receptors were highly conserved across five noctuid species

**Fig. 1:**
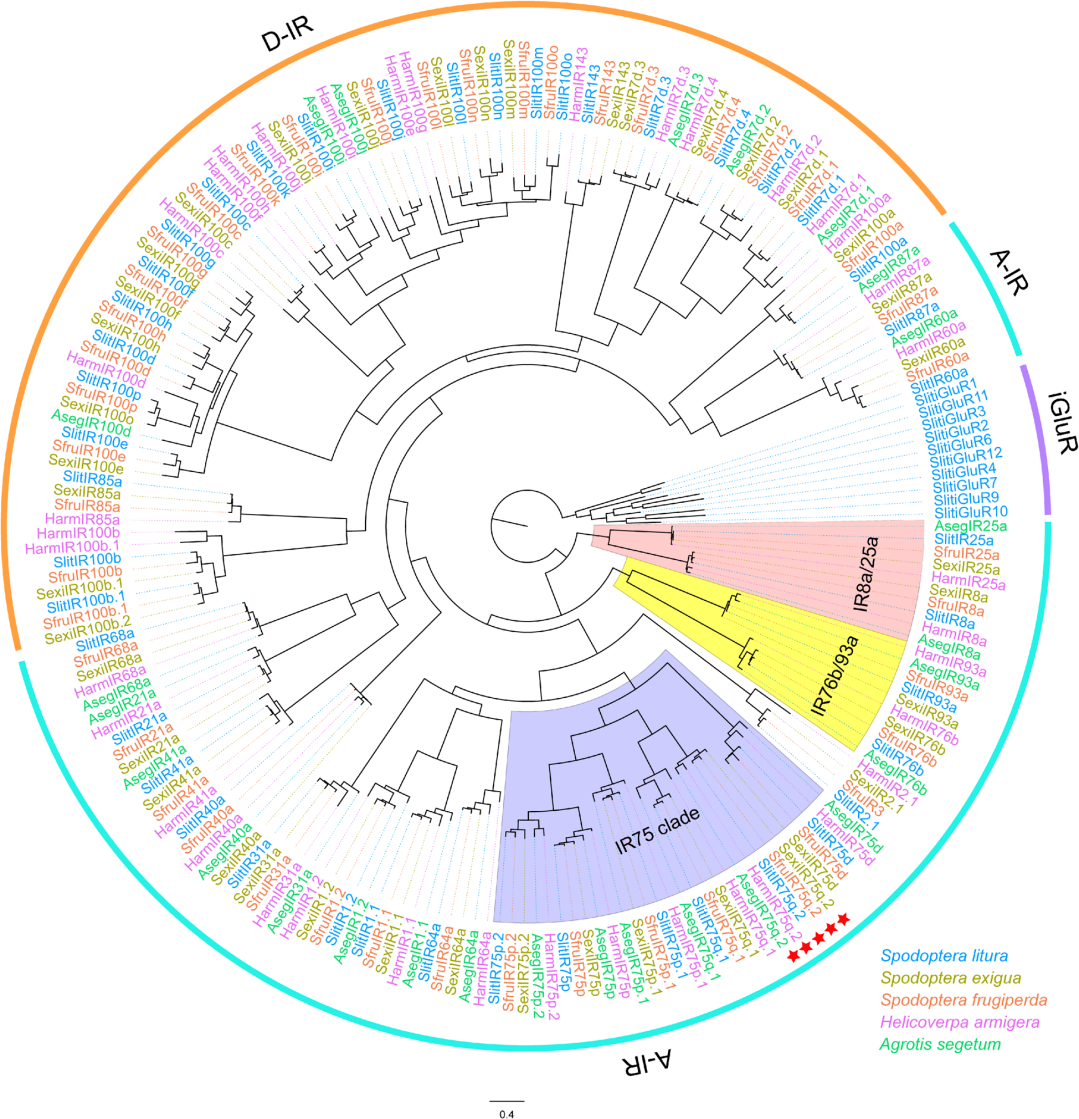
Phylogenetic analysis of ionotropic receptors (IRs) from five noctuid species (*S. litura*, *S. exigua, S. frugiperda, A. segetum* and *H. armigera*), using the maximum-likelihood method. Ten iGluRs from *S. litura* are used to root the tree

To assess the conservation of the IRs across species, we performed a phylogenetic analysis using amino acid sequences from three *Spodoptera* species and two other noctuid species. The analysis included 30 IR75 sequences, with six sequences per species reported from published studies [26, 28]. The phylogenetic analysis revealed that the IR75 receptors form a highly conserved clade, displaying a strong homologous relationship among these moths (Fig. 1).

**Fig. 2:**
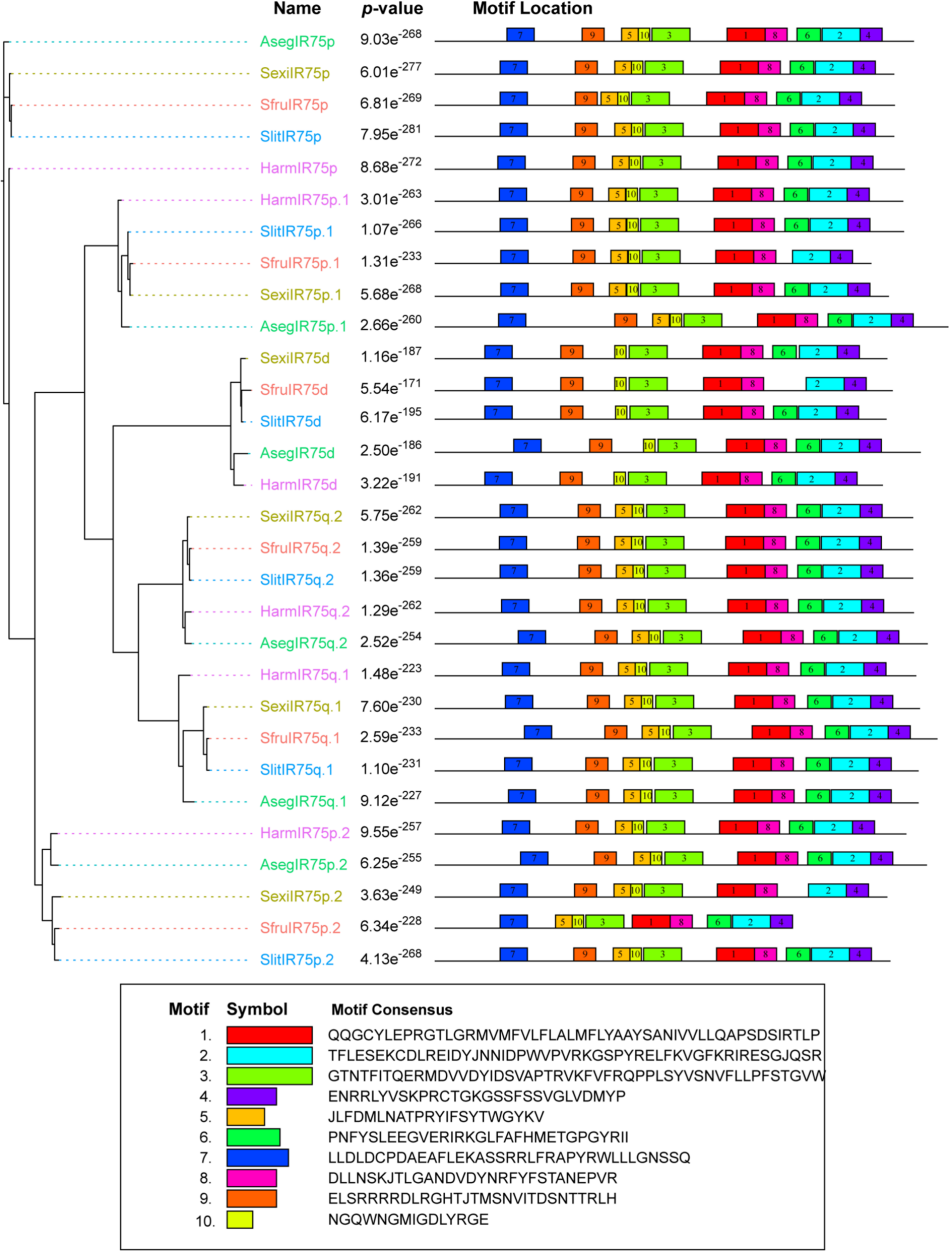
Conserved motifs of IRs in the IR75 clade of five noctuid species. Left, phylogenetic tree of the IR75 clade in five species. Slit, *S. litura*, Sexi, *S. exigua,* Sfru*, S. frugiperda,* Aseg, *A. segetum* and Harm, *H. armigera*. Right, schematic distribution of 10 conserved motifs in IRs of the IR75 clade. The colored boxes represent conserved motifs of each IR. The combined match *p*-value represents the probability that a random sequence would have position *p*-values such that their product is smaller or equal to the value calculated for the sequence under test

The conserved motifs of the IR75 clade from all five species were analyzed using the MEME Program, and top ten conserved motifs were identified. These IRs exhibited a highly consistent motif pattern, with all ten motifs present in 22 out of the 30 IRs. The motifs were arranged in nearly the same order (7–9-5–10-3–1–8–6-2–4) and were located in similar regions. Furthermore, all 10 motifs demonstrated exceptionally high conservation, with *p*-values lower than 10^–170^ (Fig. 2). Such highly conserved amino acid sequence patterns suggest that members of the IR75 clade may share structural features related to ligand recognition.

To provide a more direct comparison of sequence conservation, we further performed a multiple sequence alignment of IR75q and closely related IR75-family homologs from representative insect orders (Fig. S1). The alignment positions corresponding to the predicted ligand-binding residues Phe285/Arg290 in SlitIR75q.2 and Phe284/Arg289 in SexiIR75q.2 were marked with red asterisks. Notably, the Arg position was highly conserved among the examined IR75-family homologs, whereas the Phe position and surrounding regions showed stronger conservation within *Spodoptera* IR75q.2 orthologs and greater divergence among more distant homologs.

### IR75q.2 orthologs showed conserved expression patterns, high sequence conservation and strong purifying selection

**Fig. 3:**
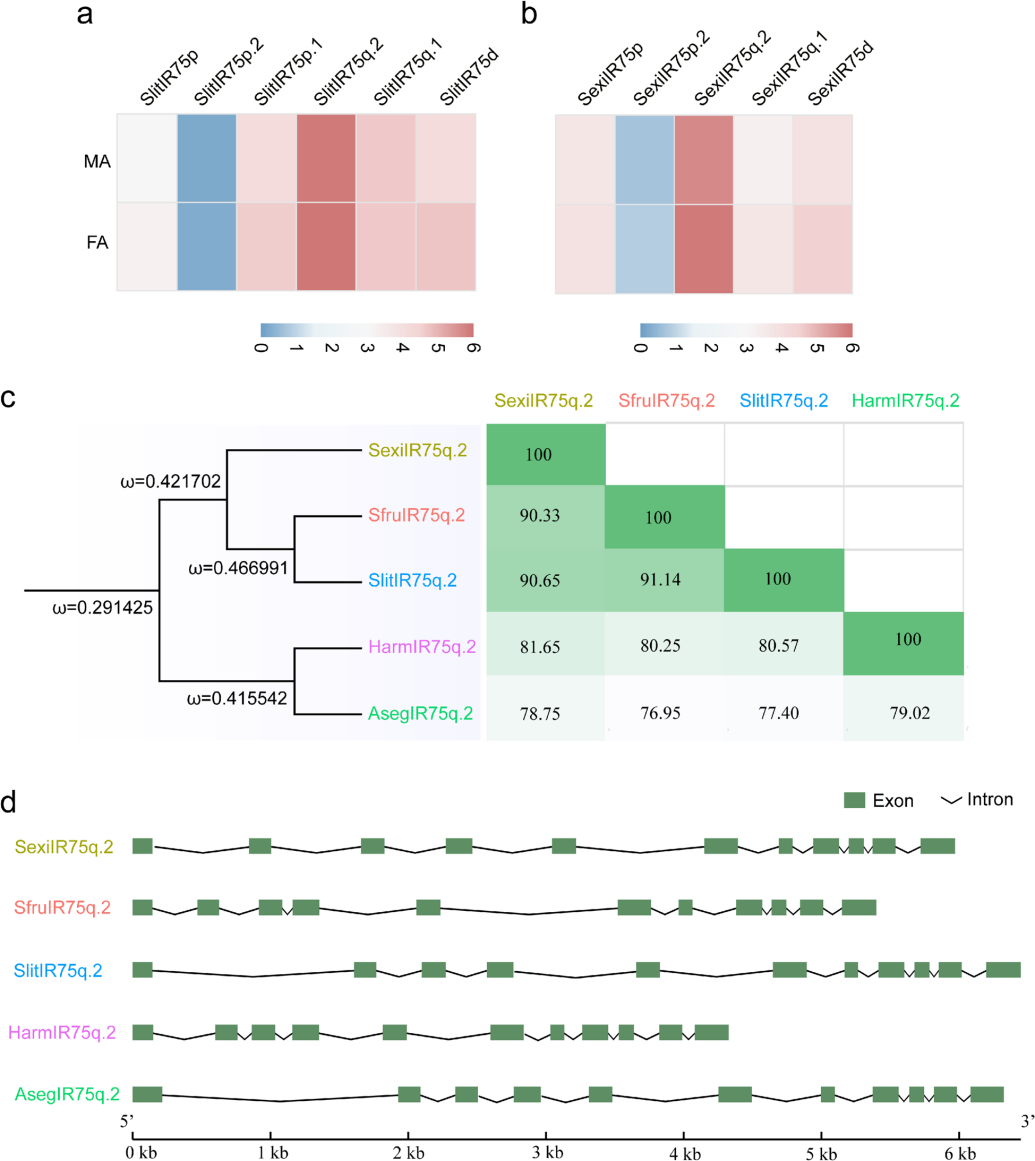
Expression profiles of IR75 clade genes in *S. litura* and *S. exigua* and conservation of IR75q.2 orthologs across five noctuid moth species. **a**, **b** Expression profiles of IRs in the IR75 clade of *S. litura* (a) and *S. exigua* (b) in female antennae (FA) and male antennae (MA). Expression levels are shown as log2(TPM + 1) values. Differential expression was analyzed using edgeR, with significance defined as FDR < 0.05 and |log_2_FC|> 1. **c** Phylogenetic relationship and pairwise amino acid sequence identity of IR75q.2 orthologs from *S. litura*, *S. exigua*, *S. frugiperda*, *A. segetum* and *H. armigera*. Pairwise amino acid sequence identities were calculated based on MAFFT alignments. Nonsynonymous/synonymous substitution rate ratios (ω) are annotated on the corresponding branches. **d** Gene structures of IR75q.2 orthologs from the five species

To provide a broader view of IR expression, RNA-seq datasets from male and female antennae of *S. litura* and *S. exigua* were analyzed to examine the expression profiles of annotated IR genes available in the genome datasets (Fig. S2). Among the IR75 clade, IR75q.2 exhibited the highest expression level in both species, with no significant sex-based differences (Fig. 3a, b). This finding supports the potential importance of IR75q.2 in antennal olfaction. The five IR75q.2 orthologs shared high amino acid sequence identity and contained the same number of exons. Notably, SlitIR75q.2 and SexiIR75q.2 shared 90.65% sequence identity (Fig. 3c). Gene structure analysis showed that both SlitIR75q.2 and SexiIR75q.2 consisted of 11 exons, whereas their ORFs were 1896 and 1893 bp in length, encoding 631 and 630 amino acids, respectively. Both proteins possessed three transmembrane domains (Fig. 3d; Fig. S3). Selection pressure analysis using PAML 4.10 showed that the branch model significantly rejected the one-ratio model (*p* < 0.05), indicating divergent selective pressures across different branches within the IR75q.2 group. However, ω values of all branches were < 1, suggesting that IR75q.2 orthologs are under strong purifying selection (Fig. 3c).

### IR75q.2 responded strongly to nonanoic acid in both *S. litura* and *S. exigua*

**Fig. 4:**
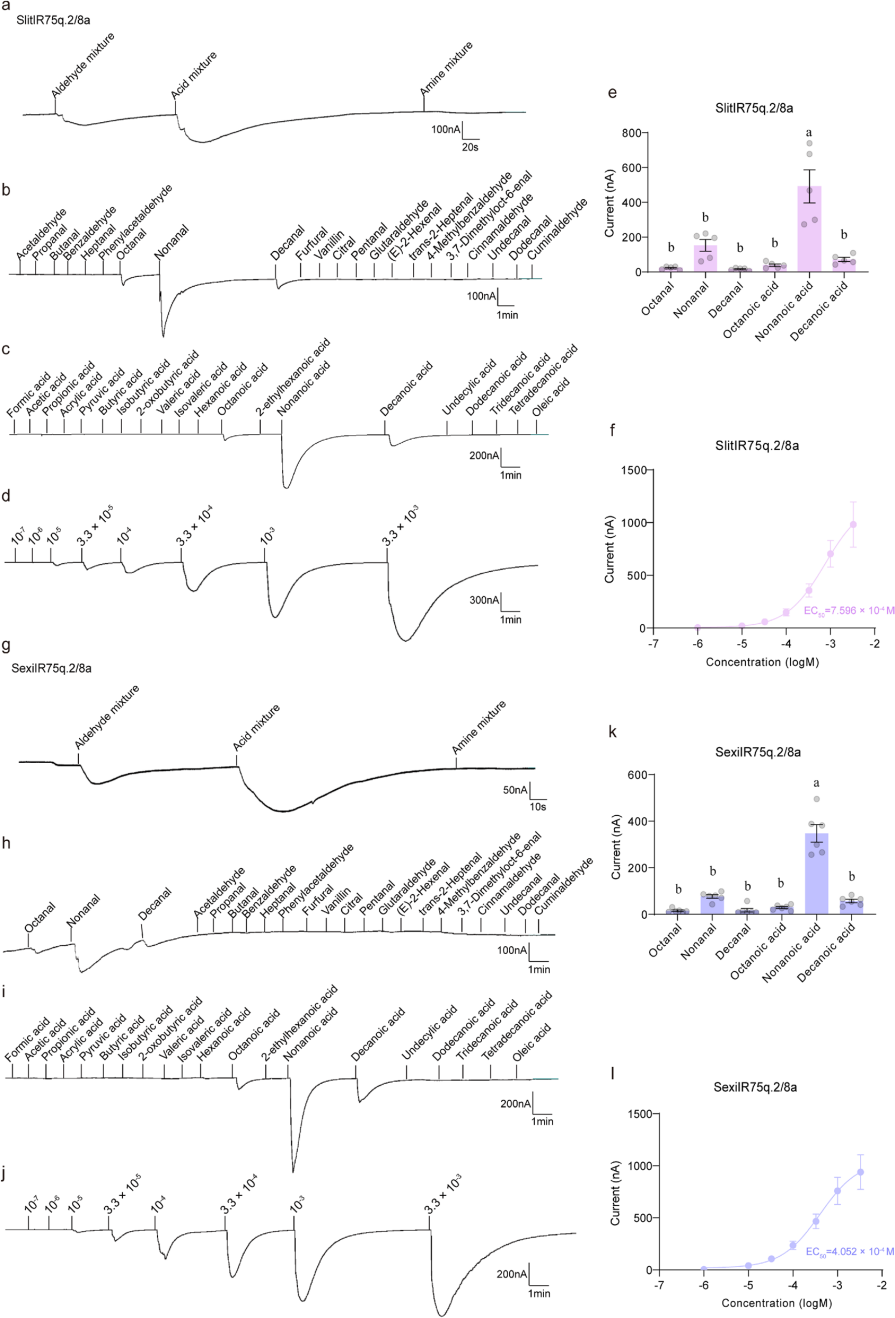
Functional characterization of SlitIR75q.2/8a and SexiIR75q.2/8a in the *Xenopus* oocyte system. **a**–**d** Representative current traces from *Xenopus* oocytes co-expressing SlitIR75q.2/8a in response to odorant mixtures, aldehydes, and acids at 10⁻^4^ M (**a**–**c**), and nonanoic acid at varying concentrations (**d**). **e** Ligand response profile of oocytes co-expressing SlitIR75q.2/8a. Data are presented as the mean ± standard error of the mean (SEM) (n = 5). **f** Dose–response curve of SlitIR75q.2/8a to nonanoic acid. Data are presented as the mean ± SEM (n = 8). **g**–**j** Representative current traces from *Xenopus* oocytes co-expressing SexiIR75q.2/8a in response to odorant mixtures, aldehydes, and acids at 10⁻^4^ M (**g**–**i**), and nonanoic acid at varying concentrations (**j**). **k** Ligand response profile of oocytes co-expressing SexiIR75q.2/8a. Data are presented as the mean ± SEM (*n* = 6). **l** Dose–response curve of SexiIR75q.2/8a to nonanoic acid. Data are presented as the mean ± SEM (*n* = 8). Concentrations in dose–response curves are plotted on a logarithmic molar scale. Different letters above the bars indicate significant differences among ligands, as determined by one-way ANOVA followed by Tukey’s multiple comparison test (*p *< 0.05)

Functional analyses of SlitIR75q.2 and SexiIR75q.2 were performed using the XOE-TEVC system. A total of 59 compounds were tested in three separate mixtures at a concentration of 10^–4^ M (Fig. 4a, g). The results showed that both IRs responded primarily to six compounds, including three acids (octanoic acid, nonanoic acid and decanoic acid) and their corresponding aldehydes (octanal, nonanal and decanal) (Fig. 4b, c, h, i). For SlitIR75q.2/8a, nonanoic acid elicited the strongest response (491.61 nA), while nonanal and decanoic acid induced moderate responses (71.90–153.00 nA), and decanal, octanal and octanoic acid triggered weaker responses (15.56–38.34 nA) (Fig. 4e). A similar response profile was observed for SexiIR75q.2/8a. Nonanoic acid induced the highest response (347.70 nA), followed by moderate responses to nonanal and decanoic acid (57.22–76.5 nA), and lower responses to octanal, decanal and octanoic acid (15.47–30.55 nA) (Fig. 4k). Furthermore, the dose–response assays for the strongest ligand, nonanoic acid revealed EC_50_ values of 7.596 × 10^–4^ M for SlitIR75q.2 (Fig. 4f) and 4.052 × 10^–4^ M for SexiIR75q.2 (Fig. 4l). Together, these results indicate that IR75q.2 from the two Spodoptera species exhibits highly similar ligand response profiles.

### IR75q.2 showed conserved ligand-binding features for nonanoic acid in both *S. litura* and *S. exigua*

**Fig. 5:**
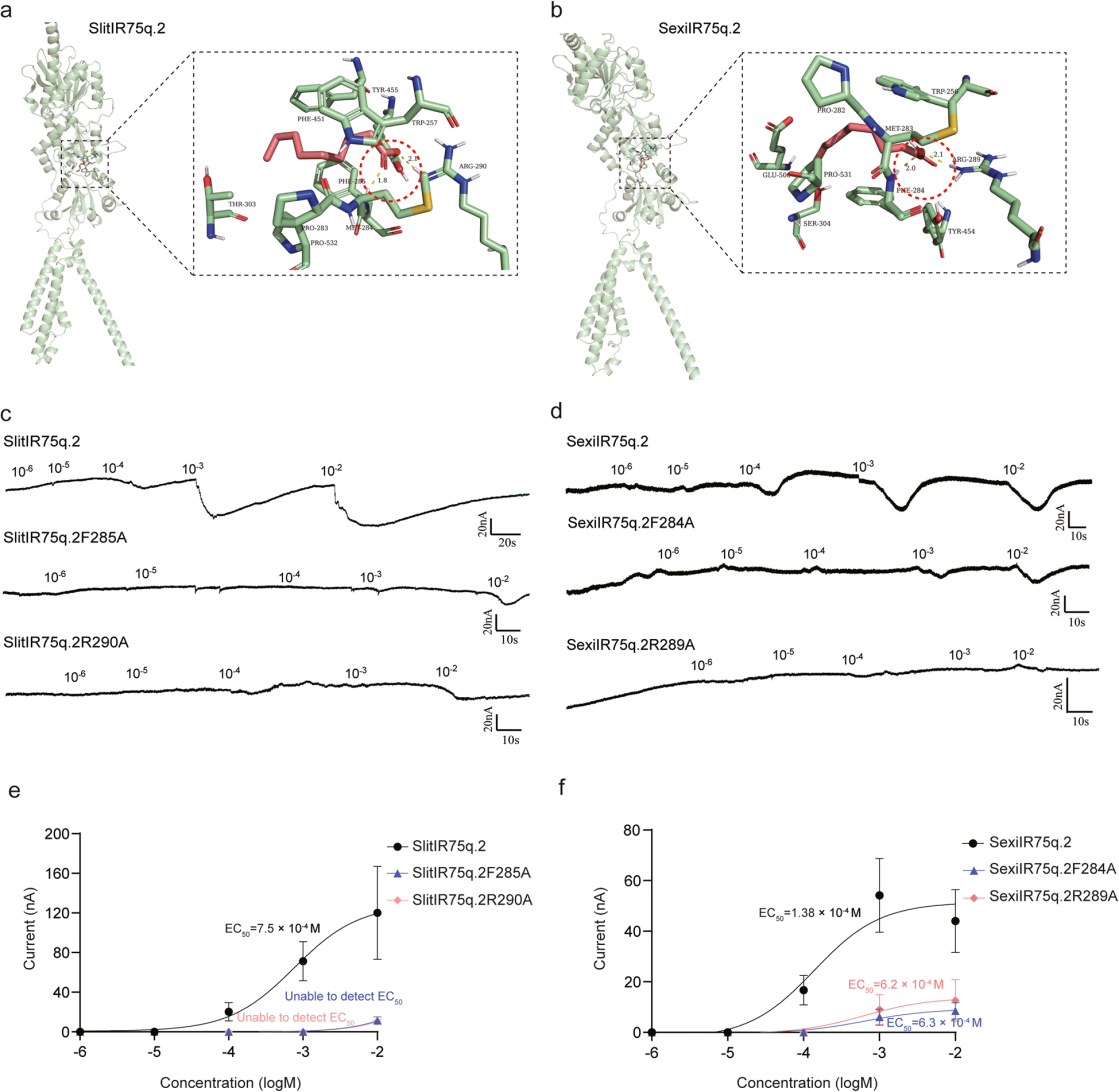
Predicted 3D structures (left) and molecular docking with nonanoic acid (right) of SlitIR75q.2 (**a**) and SexiIR75q.2 (**b**). Green sticks indicate residues within 4 Å of nonanoic acid (shown in deep salmon). The yellow dashed line (inside the red circle) represents the hydrogen bond, and the number represents the distance (Å). **c** Responses of *Xenopus* oocytes, co-expressing with SlitIR75q.2/8a and two mutant constructs to nonanoic acid at varying concentrations. (**d**) Responses of *Xenopus* oocytes, co-expressing with SexiIR75q.2/8a and two mutant constructs to nonanoic acid at varying concentrations. (**e**) Dose–response curves of SlitIR75q.2/8a and two mutant constructs expressed in *Xenopus* oocytes in response to nonanoic acid. Data are presented as the mean ± SEM (*n* = 6). **f** Dose–response curves of SexiIR75q.2/8a and two mutant constructs expressed in *Xenopus* oocytes in response to nonanoic acid. Data are presented as the mean ± SEM (*n* = 6)

To study the interaction between IRs and their strongest ligand (nonanoic acid), AlphaFold 3.0 was used to predict the 3D structures of IRs. The results showed that 91.2% and 91.1% of amino acid residues of SlitIR75q.2 and SexiIR75q.2 were located in the most favored regions (A, B, and L), respectively (Fig. S4). Further molecular docking analysis showed that the two IRs featured similar ligand-binding pockets for nonanoic acid (Fig. 5a, b). The predicted binding energy between SlitIR75q.2 and nonanoic acid was −6.04 kcal/mol, with residues Phe285 and Arg290 forming hydrogen bonds with the ligand. For SexiIR75q.2, the binding energy was −4.93 kcal/mol, with hydrogen bonds formed between nonanoic acid and residues Phe284 and Arg289. Interestingly, these Phe and Arg residues are located within the S1 domain of the LBD (Fig. S3). Additionally, 10 amino acid residues within 4 Å of nonanoic acid were conserved between SlitIR75q.2 and SexiIR75q.2 (Table S1), indicating strong conservation of key residues involved in nonanoic acid binding.

To examine the functional importance of the two amino acid residues, Phe and Arg, which were predicted to form hydrogen bonds with nonanoic acid, alanine-substitution mutants were generated by site-directed mutagenesis. cRNAs transcribed from the four mutant constructs were expressed in *Xenopus* oocytes and ligand-evoked currents were recorded by two-electrode voltage clamp. Substitution of either Phe285 or Arg290 with alanine abolished the response to nonanoic acid, precluding EC_50_ estimation (Fig. 5c; e). Similarly, in SexiIR75q.2, single alanine substitution of Phe284 or Arg289 increased the EC_50_ values to 6.3 × 10^–4^ and 6.2 × 10^–4^ M, respectively, compared with 1.38 × 10^–4^ M for intact SexiIR75q.2 (Fig. 5 d; f).

Molecular docking of SlitIR75q.2 and SexiIR75q.2 with the other five compounds (octanal, nonanal, decanal, octanoic acid, and decanoic acid) were also performed (Table S1; Fig. S5). For all five compounds, as with nonanoic acid, the two receptors shared conserved hydrogen-bonding anchors (Phe285/Arg290 in SlitIR75q.2 and Phe284/Arg289 in SexiIR75q.2) and several hydrophobic residues (e.g., Ile230/229, Trp257/256, and Pro282/282). However, IR- and ligand-specific differences were observed in the number of hydrogen bonds and hydrophobic residues.

### Nonanoic acid elicited electrophysiological responses in both species and behavioral avoidance in *S. litura*

**Fig. 6:**
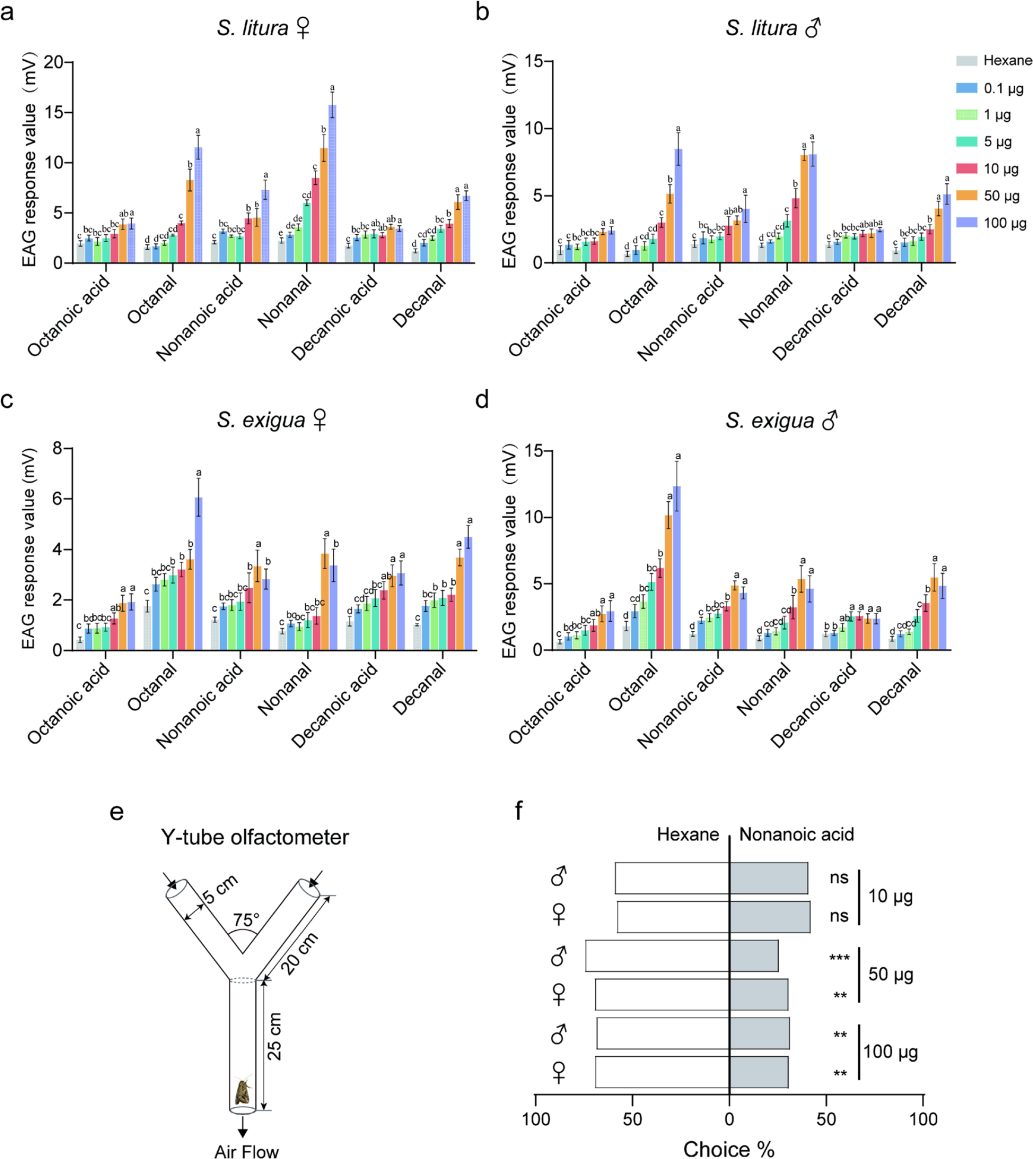
Electroantennogram (EAG) responses of *S. litura* and *S. exigua* to six compounds (**a**-**d**) and behavioral responses of *S. litura* to nonanoic acid (**e**, **f**). **e** Schematic diagram of the Y-tube olfactometer used for the behavioral assay. In a-d, different letters indicate significant difference between EAG values (mean ± SEM, n = 5–6) induced by the compound of different dosages (one-way ANOVA followed by Tukey's test, *p* < 0.05). In panel f, numbers within the panel indicate the number of replicates. Statistical significance was evaluated by *X*.^*2*^ test (**p < 0.01; ***p < 0.001)

To investigate the behavioral activity of nonanoic acid in the two *Spodoptera* moths, we conducted EAG and Y-tube olfactometer experiments. EAG assay demonstrated that adults of both *S. litura* and *S. exigua* exhibited significant responses in a dose-dependent manner to nonanoic acid and other five ligands of IR75q.2 (Fig. 6a-d; Fig. S6). Subsequent behavioral assays using nonanoic acid revealed significant repellent effects against *S. litura* at doses of 50 μg and 100 μg (Fig. 6e, f). However, *S. exigua* adults displayed extremely low locomotor activity in the Y-tube olfactometer, precluding reliable behavioral testing for this species.

## Discussion

IRs are an ancient and conserved class of receptors found in invertebrates, initially discovered in *Drosophila* [8]. They play a crucial role in insect chemosensory systems, enabling the detection of a variety of environmental chemical signals [17, 30, 39, 41, 42]. In the present study, we demonstrate that IR75q.2 orthologs exhibit significant structural and functional conservation in two *Spodoptera* species. Furthermore, we identified specific residues involved in interactions with nonanoic acid and other ligands. These results provide valuable insights into the functional evolution of the IR75 clade in Lepidoptera and highlight potential targets for developing novel pest management strategies.

Phylogenetic analysis showed that IR75s formed the largest homologous clade among the IRs in lepidopteran species. This result is consistent with previous studies [26, 28, 41], suggesting that these IR genes play an important role in these insects. The conservation of IR75s is further supported by our motif analysis, which showed that IR75s retain a highly conserved structure and motifs across the five noctuid species. Such conservation suggests similarity in the structural frameworks of these receptors, although ligand specificity may still differ among lineages. Moreover, evolutionary analysis based on dN/dS ratios revealed that IR75q.2 genes are under strong purifying selection, highlighting their importance in maintaining functional stability across species.

Among the IR75 clade genes, IR75q.2 showed the highest antennal expression in both sexes of *S. litura* and *S. exigua*. As the primary olfactory organs in insects, antennae are responsible for detecting and interpreting chemical signals from the environment. The high expression of IR75q.2 suggests a potential role in detecting specific chemical cues of ecological importance. Fatty acids such as octanoic acid and decanoic acid are produced during the process of nectar fermentation and function as key indicators of nectar quality. Notably, octanoic acid produced in the late fermentation stage mediates the avoidance behavior of *H. armigera,* as it significantly reduces mating rates and egg hatching rates [42]. Our functional assays demonstrated that IR75q.2 from both *S. litura* and *S. exigua* responded to octanoic acid, nonanoic acid, and decanoic acid, suggesting its role in sensing fermentation-derived fatty acids from nectars and other substrates.

The role of IR75q.2 in fatty acid detection is consistent with the known function of the IR75 subfamily in insect acid sensing. In *Drosophila*, IR75 receptors such as IR75a, IR75b, and IR75c have been associated with the detection of carboxylic acids, with IR8a acting as an essential co-receptor in acid-sensitive IR pathways [24]. Together with previous findings on *S. frugiperda* IR75q.2, our results further support the conserved involvement of IR75q.2 in the detection of medium-chain fatty acids in *Spodoptera* moths, while adding comparative evidence from two additional species and identifying conserved residues that contribute to nonanoic acid recognition [40]. Interestingly, the ligands detected by IR75q.2 partly overlap with those reported for some odorant receptors. In particular, nonanal, one of the aldehydes that activated SlitIR75q.2 and SexiIR75q.2 in this study, has been reported to attract females of *S. frugiperda* and *S. litura*, and a conserved odorant receptor lineage represented by SfruOR47 and SlituOR9 was shown to be narrowly tuned to nonanal [45]. This finding suggests that aldehydes such as nonanal may be detected through both OR- and IR-mediated olfactory pathways in *Spodoptera* moths. The coexistence of these two pathways for partially overlapping odorants may have important biological significance. Many ORs have been shown to mediate the detection of plant-derived neutral volatiles, including aldehydes, alcohols, esters and terpenoids, whereas antennal IRs are often associated with the detection of acids, amines and other ecologically relevant polar cues. Thus, ORs and IRs may not simply duplicate each other, but may instead provide complementary information. OR-mediated detection of nonanal may contribute to attraction to host-related plant volatiles, whereas IR75q.2-mediated detection appears to be biased toward medium-chain fatty acids, especially nonanoic acid, which elicited the strongest receptor response and avoidance behavior in this study. Such parallel coding may help moths discriminate chemically related compounds with different behavioral valences, broaden the detectable chemical space, and improve the robustness of olfactory-guided host selection and avoidance decisions.

Although IR75q.2 orthologs in *S. litura* and *S. exigua* responded to C8–C10 fatty acids and their corresponding aldehydes, they responded primarily to the aversive compound nonanoic acid, consistent with the response profile of the IR75q.2 ortholog in *S. frugiperda* [40], further confirming the functional conservation of IR75q.2s in *Spodoptera* species. However, whether this function and ligand response profile of IR75q.2s are broadly conserved in Noctuidae remains unclear, as available functional evidence from *A. segetum* does not fully support a universally conserved pattern. Although phylogenic and motif analyses suggest structural conservation of the IR75q.2-related lineage in Noctuidae, ligand specificity may differ among lineages. Very recently, the IR75q.1 group was reported to respond primarily to the aversive octanoic acid and weakly to nonanoic acid, which is conserved in noctuid species (*H. armigera, Mythimna separata, A. segetum* and *S. frugiperda*), but not in non-noctuid species (*Bombyx mori* and *Cydia pomonella*) [42]. Considering together with the close phylogenetic relation, these findings suggest differentiation in ligand preference between the IR75q.1 and IR75q.2 groups. Overall IR75q.1s and IR75q.2s appear to differ in their preferred fatty acid ligands, although the exact response pattern may vary among lineages, supporting the idea that subfunctionalization after gene duplication has played an important role in the evolution of ligand specificities of the acid‑sensing IRs in Lepidoptera [42].

Our structural modeling and molecular docking analysis provided insights into the molecular interactions between IR75q.2 and its ligands. The predicted strong binding affinity between IR75q.2 orthologs and nonanoic acid indicates a favorable interaction within the ligand-binding pocket. Notably, Phe285/284 and Arg290/289 were predicted to form hydrogen bonds with nonanoic acid, and site-directed mutagenesis further supported the importance of these residues in ligand recognition in *S. litura* and *S. exigua*. Beyond identifying candidate binding residues, these results provide a possible structural explanation for the conserved nonanoic acid sensitivity of IR75q.2 and suggest that subtle changes in key binding-pocket residues may have contributed to the evolutionary divergence of ligand specificity among acid-sensing IRs in Lepidoptera.

## Materials and methods

### Insect rearing and tissue collection

*S. litura and S. exigua* were maintained under controlled laboratory conditions of 27 ± 1 °C, 65 ± 5% relative humidity, and a 14: 10 h light: dark photoperiod. Larvae were provided with an artificial diet [46]. The pupae were sexed and transferred to separate cages until eclosion. The moths were reared on a 10% honey solution. Fifty antennae from 2-d-old virgin males and females were collected between the 6th to 8th hours of the dark period. All the samples were snap-frozen in liquid nitrogen and stored at − 80 °C until use.

### RNA extraction, cDNA synthesis and molecular cloning

Total RNA was isolated separately from the antennae of *S. litura* and *S. exigua* using the an SV total RNA isolation system (Promega, Madison, USA), according to the manufacturer’s instructions. First-strand cDNA was generated with HiScript® III RT SuperMix for qPCR (+ gDNA wiper) (Vazyme, Nanjing, China), following the manufacturer’s protocol. The cDNA was stored at − 20°C. To obtain the target gene sequences, the previously reported amino acid sequences of *IR8a* and *IR75q.2* [26, 27] were used as queries to search the genomes of *S. litura* and *S. exigua* by TBLASTN [47]. Candidate coding regions were further examined with ORF Finder (https://www.ncbi.nlm.nih.gov/orffinder/) to define the open reading frames (ORFs). Specific primers for gene cloning and cRNA synthesis were designed with Primer Premier 5.0 (PREMIER Biosoft International, CA, USA) [48] and CE design (Vazyme, Nanjing, China), respectively (Table S2). PCR was performed in a 25 μL reaction system containing 2 × Phanta®Max Master Mix (super fidelity) according to the manufacturer's instructions (Vazyme, Nanjing, China). Amplicons of the expected size were cloned into the pEASY®-Blunt3 Cloning Vector (TransGen Biotech, Beijing, China) and sequenced by GENERAL BIOL Company (Chuzhou, China).

### Sequence, phylogenetic and evolutionary analysis

Previously reported amino acid sequences of IR75q.2 from five species of Noctuidae (*S. litura*, *S. exigua, S. frugiperda, Agrotis segetum*, and *Helicoverpa armigera*) reported in the literature [26, 28, 41], were aligned by DNAMAN version 8 (Lynnon LLC, San Ramon, USA). Putative transmembrane domains of IR75q.2 were predicted by TOPCONS (https://topcons.net/). For phylogenetic analysis, IR amino acid sequences from the five noctuid species were aligned using MAFFT version v7.407 [49] with default parameters. The phylogenetic tree was constructed using IR amino acid sequences from the five aforementioned species. The maximum-likelihood tree was conducted by FastTree v2.1.10 [50]. The final tree was visualized and annotated using FigTree version 1.4.4 [51]. The conserved motifs among the IR75 sequences were analyzed using the MEME Program, with the maximum number of motifs set to ten [52]. For broader homologous comparison, IR75q and closely related IR75-family homologs from representative insect orders were aligned using MAFFT and visualized with Jalview. The alignment positions corresponding to the predicted ligand-binding residues were manually annotated.

The coding sequences (CDS) of *IR75q.2* from five species were aligned to their respective genomes to obtain the intron and exon sequence information. Subsequently, the gene structures were visualized using the GSDS 2.0 web servers [53]. The selective constraints on IR75q.2 s were evaluated under a maximum-likelihood method [54] using the Codeml program in the PAML 4.10 package [55], with ω (dN/dS) used as a measure of selection pressure.

### The IR gene expression analysis

Publicly available antennal RNA-seq datasets were used to examine IR expression profiles in male and female adults. The datasets (*S. litura*: SRR5248293 and SRR5248294; *S. exigua*: SRR15212580 and SRR15212581) were obtained from the NCBI SRA database (https://www.ncbi.nlm.nih.gov/sra). Clean paired-end reads were mapped to the corresponding reference genomes *of S. litura* (InsectBase ID: IBG_00716) and *S. exigua* (InsectBase ID: IBG_00714) using HISAT2 version 2.2.1 [56]. The reference genome and gff3 annotation files were downloaded from InsectBase (http://v2.insect-genome.com) [57]. The expression abundance was calculated as transcripts per million (TPM) using an R script. Differential expression analysis was performed with the edgeR package [58]. Genes were considered significantly differentially expressed if they met the criteria of a false discovery rate (FDR) less than 0.05 and |log_2_FC| greater than 1.

### Functional analysis of IR75q.2 using Xenopus oocyte expression and two-electrode voltage clamp (XOE-TEVC) system

Functional assays were performed in *Xenopus* oocyte following previously reported protocols [40, 59]. The complete CDSs of *IR75q.2* and *IR8a* were subcloned into the pGH19 expression vector through the appropriate restriction enzyme sites. The cRNAs were synthesized using mMESSAGE mMACHINE® T7 Kit (Thermo Fisher Scientific, Waltham, USA). Mature healthy oocytes were digested with 2 mg/mL of collagenase S-1 in Ca^2+^-free standard oocyte saline (SOS) buffer containing 100 mM NaCl, 2 mM KCl, 1 mM MgCl_2_, and 5 mM HEPES, pH 7.6, for 1–2 h at room temperature. Oocytes were microinjected with 27.6 ng of *IR75q.2* and *IR8a* cRNA and maintained for 3–5 days at 16 °C in sterile SOS medium, consisting of Ca^2+^-free SOS buffer supplemented with 1.8 mM CaCl_2_, 5% dialyzed horse serum, and 2.5 mM sodium pyruvate. Test compounds listed in Table S3 were dissolved in DMSO to prepare 0.1 M stock solutions and stored at − 20°C. Before the recordings were taken, the stock solutions were diluted to the required concentrations in the SOS recording solution. The cell currents induced by the chemicals were recorded with a TEVC. Data acquisition and analysis were performed with Axon Digidata 1440 A (AutoMate Scientific, Inc., Berkeley, USA) and CLAMP 10.4 software (Axon Instruments Inc., San Jose, USA).

### Electroantennogram (EAG) recordings

The compounds (octanoic acid, octanal, nonanoic acid, nonanal, decanal and decanoic acid) were prepared in hexane at different concentrations. A 10 μL aliquot was loaded on a filter paper (2.5 × 0.8 cm), and hexane alone served as the solvent control. After solvent evaporation for 3 min, the filter paper was placed into a Pasteur tube for the EAG stimulation. EAG recordings were performed as described previously [59], using the EAG instrument and related software (SyntechR, Germany). Antennae of 3-day-old virgin male and female moths were used for recording. Each compound was measured with 6 biological replicates in both *S. litura* and *S. exigua*.

### Y-tube olfactometer bioassays

The behavioral response of 3-d-old virgin males and females *S. litura* adults to nonanoic acid were tested in a Y-tube olfactometer. The main arm of the Y-tube was 20 cm in length, and each side arm was 15 cm long, with an inner diameter of 5 cm. Bioassays were conducted at 26 ± 1 °C, under dim red illumination provided by a flashlight positioned above the Y-tube junction.

Nonanoic acid was dissolved in hexane to obtain test solutions at concentrations of 1, 5, and 10 μg/μL. A total of 10 μL of nonanoic acid solution or hexane control was applied to a filter paper strip (2.5 × 0.8 cm), and the strips were placed separately in the two lateral arms. The airflow was adjusted to 0.6 L/min in each arm and monitored with an atmospheric sampler (QC-1s, Beijing Institute of Labor Protection Science, Beijing, China). Virgin females and males were tested individually. Each moth was introduced individually at the base of the main arm and observed for 5 min. Individuals who did not enter either lateral arm during the observation period were scored as having no choice. A response was recorded when a moth crossed the midpoint of one lateral arm and remained there for at least 15 s. To minimize positional bias, the olfactometer was rotated 90° after every 10 months. At the end of each experimental day, the apparatus was rinsed with ethanol and air-dried. At least 60 virgin individuals were tested for each odor dose, and each moth was used only once.

### The 3D structure prediction, molecular docking and site-directed mutagenesis

The 3D structures of the IR75q.2s were predicted using AlphaFold 3.0 [60], and assessed using the PROCHECK server (https://saves.mbi.ucla.edu/). The 3D structure of nonanoic acid (PubChem CID: 8158) was retrieved from PubChem (https://pubchem.ncbi.nlm.nih.gov/). Molecular docking was conducted using AutoDock Vina 1.1.2 [61]. Prior to docking, the IR protein structure and ligand coordinates were prepared using AutoDock 4.2.6 [62] and Open Babel 2.3.2 [63]. The results were analyzed by Autodock tools, and docking models were imported into PyMol 3.0.4 (Schrödinger, LLC, New York, USA) for visualization. Based on the docking results, Phe284 and Arg289 in SexiIR75q.2 and Phe285 and Arg290 in SlitIR75q.2 were individually substituted with alanine using the Fast Mutagenesis System (FM111, TransGen Biotech) with the primers listed in Table S2. cRNAs from the four mutant plasmids were synthesized and expressed in *Xenopus* oocytes, and their responses to ligands were measured by two-electrode voltage clamps, as described in the XOE-TEVC section above.

## Supplementary Information

Supplementary Material 1.

## Data Availability

The data supporting the findings of this study are available within the article and its supplementary information files.
