# Supplementary material for: Functional conservation of IR75q.2 in the recognition of volatile acids and aldehydes in two Spodoptera species
Source: Crop Health. 2026 Jul 21;4(1):20. doi: 10.1007/s44297-026-00082-7 (PMC13388880; doi:10.1007/s44297-026-00082-7)
Supplement: Supplementary file 1 — Supplementary Material 1. [file 44297_2026_82_MOESM1_ESM.docx]

**Table S1 Prediction of key amino acid residues and binding energy during the docking of SlitIR75q.2 and SexiIR75q.2 with different ligands.**

| **Ligands** | **SlitIR75q.2** | |  | **SexiIR75q.2** | |
| --- | --- | --- | --- | --- | --- |
|  | **Binding energy (kcal/mol)** | **Key residue** |  | **Binding energy (kcal/mol)** | **Key residue** |
| **Octanal** | -5.06 | ILE230, TRP257, PRO283, MET284, PHE285, ARG290, THR303, PRO532 |  | -5.17 | ILE229, TRP256, PRO282, MET283, PHE284, ARG289, THR302, PHE450, TYR454, PRO531 |
| **Nonanal** | -5.47 | ILE230, TRP257, PRO283, MET284, PHE285, ARG290, THR303, SER305, GLU501, PRO532 |  | -5.37 | ILE229, TRP256, PRO282, MET283, PHE284, ARG289, THR302, PHE450, TYR454, PRO531 |
| **Decanal** | -5.67 | ILE230, TRP257, PRO283, MET284, PHE285, ARG290, THR303, PHE451, PRO532 |  | -5.68 | ILE229, TRP256, GLY257, TYR258, PRO282, MET283, PHE284, ARG289, THR302, PHE450, TYR454, PRO531 |
| **Octanoic acid** | -5.94 | ILE230, TRP257, TYR259, PRO283, MET284, PHE285, ARG290, THR303, TYR455, PRO532 |  | -6.1 | ILE229, TRP256, GLY257, TYR258, PRO282, MET283, PHE284, ARG289, THR302, PHE450, TYR454, PRO531 |
| **Decanoic acid** | -6.18 | ILE230, TRP257, TYR259, PRO283, MET284, PHE285, ARG290, THR303, SER305, TYR455, GLU501, PRO532 |  | -6.94 | TRP256, TYR258, PRO282, MET283, PHE284, ARG289, THR302, SER304, TYR454, GLU500, PRO531 |

Note: The underlined residues are the residues forming hydrogen bonds with the ligands.

**Table S2 Primers used in this study.‍**

| Primer name | Sequence (5´- 3´) |
| --- | --- |
| **For gene cloning** | |
| SlitIR8a-F | ATGTCAATTTATTATTTATTTTTG |
| SlitIR8a-R | CTAAGGCCTATAATCCTTTG |
| SlitIR75q.2-F | ATGCGGAAGATAATATTTATT |
| SlitIR75q.2-R | TTAATCGTAATTTTCCTCTTT |
| SexiIR8a-F | ATGTCAATTTATTATTTATTTTTGT |
| SexiIR8a-R | TTAAGGCCTATAATCTTTGG |
| SexiIR75q.2-F | ATGGAAAAGGCGATATGGT |
| SexiIR75q.2-R | TTAATCGTAATTTTCGTCTTTTA |
| **For cRNA synthesis primers** | |
| SlitIR8a-EcoRI-F | ATTCCCCGGGGATCC***GAATTC***ATGTCAATTTATTATTTATTTTTG |
| SlitIR8a-XbaI-R | TCGGCGATCGGGCCC***TCTAGA***CTAAGGCCTATAATCCTTTG |
| SlitIR75q.2-EcoRI-F | ATTCCCCGGGGATCC***GAATTC***ATGCGGAAGATAATATTTATT |
| SlitIR75q.2-XbaI-R | TCGGCGATCGGGCCC***TCTAGA***TTAATCGTAATTTTCCTCTTT |
| SexiIR8a-EcoRI-F | ATTCCCCGGGGATCC***GAATTC***ATGTCAATTTATTATTTATTTTTGT |
| SexiIR8a-XbaI-R | TCGGCGATCGGGCCC***TCTAGA***TTAAGGCCTATAATCTTTGG |
| SexiIR75q.2-EcoRI-F | ATTCCCCGGGGATCC***GAATTC***ATGGAAAAGGCGATATGGT |
| SexiIR75q.2-XbaI-R | TCGGCGATCGGGCCC***TCTAGA***TTAATCGTAATTTTCGTCTTTTA |
| **For Site-directed Mutagenesis** | |
| SlitIR75q.2-F285AF | GGGGGGTCTCCAATGGCCTTCACGTTTGAA |
| SlitIR75q.2-F285AR | CATTGGAGACCCCCCAATTTCAACTTCGCC |
| SlitIR75q.2-R290AF | TTCTTCACGTTTGAAGCAGTATCGATAGTC |
| SlitIR75q.2-R290AR | TTCAAACGTGAAGAACATTGGAGACCCCCC |
| SexiIR75q.2-F284AF | GGAGGATCTCCAATGGCCTTCACATTTGAG |
| SexiIR75q.2-F284AR | CATTGGAGATCCTCCAATTTCAACTTCACC |
| SexiIR75q.2-R289AF | TTCTTCACATTTGAGGCAGTGTCAATAGTC |
| SexiIR75q.2-R289AR | CTCAAATGTGAAGAACATTGGAGATCCTCC |
| Note: In primers for cRNA synthesis, the Italic sequences (*GAATTC* and *TCTAGA*) are the restriction sites of *Eco*RI and *Xba*I, respectively; and the underlined sequences are homologs to the sequences of pGH19. ‍ | |

**Table S3 Compounds used for the functional characterization of IR75q.2.**

| **Number** | **Name** | **CAS No.** |
| --- | --- | --- |
| 1 | Formic acid | 64-18-6 |
| 2 | Acetic acid | 64-19-7 |
| 3 | Propionic acid | 79-09-4 |
| 4 | Acrylic acid | 79-10-7 |
| 5 | Butyric acid | 107-92-6 |
| 6 | Pyruvic acid | 127-17-3 |
| 7 | Isobutyric acid | 79-31-2 |
| 8 | Valeric acid | 109-52-4 |
| 9 | Isovaleric acid | 503-74-2 |
| 10 | 2-oxobutyric acid | 600-18-0 |
| 11 | Decanoic acid | 334-48-5 |
| 12 | Nonanoic acid | 112-05-0 |
| 13 | 2-ethylhexanoic acid | 149-57-5 |
| 14 | Octanoic acid | 124-07-2 |
| 15 | Hexanoic acid | 142-62-1 |
| 16 | Oleic acid | 112-80-1 |
| 17 | Tetradecanoic acid | 544-63-8 |
| 18 | Tridecanoic acid | 638-53-9 |
| 19 | Dodecanoic acid | 143-07-7 |
| 20 | Undecylic acid | 112-37-8 |
| 21 | acetaldehyde | 75-07-0 |
| 22 | Propanal | 123-38-6 |
| 23 | Butanal | 123-72-8 |
| 24 | benzaldehyde | 100-52-7 |
| 25 | Heptanal | 111-71-7 |
| 26 | Phenylacetaldehyde | 122-78-1 |
| 27 | Octanal | 124-13-0 |
| 28 | Decanal | 112-31-2 |
| 29 | Nonanal | 124-19-6 |
| 30 | Furfural | 98-01-1 |
| 31 | Vanillin | 121-33-5 |
| 32 | Citral | 5392-40-5 |
| 33 | Pentanal | 110-62-3 |
| 34 | Glutaraldehyde | 111-30-8 |
| 35 | (E)-2-Hexenal | 6728-26-3 |
| 36 | trans-2-Heptenal | 18829-55-5 |
| 37 | 4-Methylbenzaldehyde | 104-87-0 |
| 38 | 3,7-Dimethyloct-6-enal | 106-23-0 |
| 39 | Cinnamaldehyde | 104-55-2 |
| 40 | Undecanal | 112-44-7 |
| 41 | Dodecanal | 112-54-9 |
| 42 | Cuminaldehyde | 122-03-2 |
| 43 | 1,2-Diaminopropane | 78-90-0 |
| 44 | Butyronitrile | 109-74-0 |
| 45 | Cyclobutanamine | 2516-34-9 |
| 46 | Butylamine | 109-73-9 |
| 47 | 1,4-Butanediamine | 110-60-1 |
| 48 | 1,5-Diaminopentane | 462-94-2 |
| 49 | Amylamine | 110-58-7 |
| 50 | Methylbutylamine | 110-68-9 |
| 51 | 3-Aminopentane | 616-24-0 |
| 52 | Tetrameen | 110-18-9 |
| 53 | 1,6-Hexanediamine | 124-09-4 |
| 54 | Spermidine | 124-20-9 |
| 55 | 2-PhenylethylaMine | 64-04-0 |
| 56 | 1,8-Diaminooctane | 373-44-4 |
| 57 | Decylamine | 2016-57-1 |
| 58 | Spermine | 71-44-3 |
| 59 | 2-Pentylpyridine | 2294-76-0 |

**Fig. S1** Multiple sequence alignment of putative IR75q and closely related IR75-family homologs from representative insect orders. Amino acid sequence alignment of selected IR75q/IR75q-like proteins from representative insects, including *Drosophila melanogaster* and *Anopheles gambiae* (Diptera), *Tribolium castaneum* (Coleoptera), *Apis mellifera* (Hymenoptera), *Aphis glycines* (Hemiptera), and lepidopteran species including *Bombyx mori*, *Helicoverpa armigera*, *Agrotis segetum*, *Spodoptera frugiperda*, *Spodoptera litura*, and *Spodoptera exigua*. Multiple sequence alignment was performed using MAFFT and visualized with Jalview. Conserved amino acid residues are highlighted according to the Jalview colouring scheme. Red asterisks indicate the alignment positions corresponding to the predicted ligand-binding residues Phe285/Arg290 in SlitIR75q.2 and Phe284/Arg289 in SexiIR75q.2.

Fig. S2 Expression profiles of all identified ionotropic receptor (IR) genes in the antennae of *S. litura* and *S. exigua*. Heatmaps show log2(TPM + 1) values of IR genes in male antennae (MA) and female antennae (FA).

**Fig. S3** Sequence alignment of IR75q.2 orthologs from five species. The amino acid identity of the five sequences is 86.60%. Binding domain lobes (S1 and S2), the pore loop region (P), the transmembrane domains (M1, M2, and M3) were marked by different colored headings. The residues predicted to form hydrogen bonds with nonanoic acid were highlighted with a red box.

**Fig. S4** Evaluation of the 3-D structure for SlituIR75q.2 (A) and SexiIR75q.2 (B) using PROCHECK Ramachandran plots, showing >90% residues in the most favored regions. A, B and L (red) represent the residues in the most favored regions, whereas a, b, l and p (bright yellow) represent residues in additional allowed regions, and ~a, ~b, ~l and ~p (light yellow) represent residues in generously allowed regions.

**Fig. S5** Binding modes and key residues of two IR75q.2s to the five components.

**Fig. S6** Representative EAG traces of *S. litura* and *S. exigua* antennae in response to active ligands. Representative electroantennogram recordings showing the antennal responses of male and female adults of *S. litura* and *S. exigua* to the tested active ligands at a dose of 100 μg. These representative traces correspond to the quantitative EAG results presented in Fig. 6.
